# The formation of the Freudian universal symbol: a historical perspective

**DOI:** 10.3389/fpsyg.2025.1682371

**Published:** 2025-10-10

**Authors:** Chuansong Huo, Fei Ju

**Affiliations:** 1 School of Humanity, Tongji University, Shanghai, China; 2 Laboratory of Psychopathology and Psychoanalysis, Graduate School of Human and Environmental Studies, Kyoto University, Kyoto, Japan

**Keywords:** symbolism, psychoanalysis, Freudian symbol, symbol formation, dream-analysis

## Abstract

This paper emphasizes the significance of the Freudian universal symbol by examining the controversial period from 1909 to 1917, when psychoanalysts increasingly turn their attention to cultural elements such as myth and language. In the early years of psychoanalysis, Freud does not actively encourage the investigation of symbolism. Instead, it is his colleagues and disciples that contribute to shaping the notion of universal symbolism—namely, the idea that symbols derive from a phylogenetic heritage and possess constant meanings that operate unconsciously and trans-subjectively. The exploration of symbolism during this period serves to bridge psychoanalysis and folk psychology, thereby expanding its intellectual influence. However, the phylogenetic assumption underlying the Freudian universal symbol generates considerable controversy within this field. In this paper, we propose that it is suitable to dispense with this Lamarckian position while retaining the comparative paradigm of investigating dreams, myths, and language.

## 1 Introduction

In psychoanalysis, symbolism constitutes a crucial yet controversial field. Some relevant disciplines often regard psychoanalytic practice as a form of symbolic method, one that interprets patients' dreams as bearing persistent—mostly sexual—significance. For example, after Freud presents his innovative theories at Clark University, William James has a brief conversation with him. James later remarks skeptically: “His dream theory totally can't persuade me, and ‘symbol' is apparently a dangerous method” (Scull, 2015, p. 330). Beyond Freud's contemporaries, subsequent scholars have likewise tended to treat psychoanalysis as a form of symbolism. In *The Effectiveness of Symbols*, Claude Lévy-Strauss reconstructs the concept of the unconscious by comparing psychoanalytic practice with shamanic healing rituals. For him, “It (The unconscious) is reducible to a function—the symbolic function” (Lévy-Strauss, 1963, p. 203). He reduces the central psychoanalytic concept “unconscious” to a specific structural function that imposes laws upon the unordered instinctual impulse. Ricoeur (1970) also situates symbolism at the core of his research of Freud, in which he proposes that symbol is the expression of double meaning, thereby naturally establishing the affinity between symbolism and interpretation. These adjacent subjects all regard symbolism as an essential method of psychoanalysis and underscore its importance for psychoanalytic study. But what of the stance and discourse of psychoanalysts themselves? Do they also accord symbols a central place in their armamentarium?

**Figure 1:**
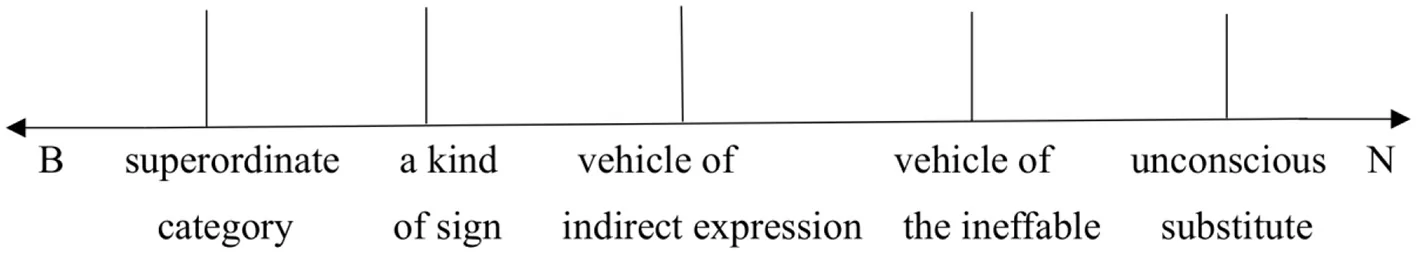
The five categories of symbol.

Among psychoanalytic literatures, the term *symbol* is employed in varying ways. Petocz (1999, p. 10–14) identifies five distinct meanings of symbol along a broad-to-narrow continuum, which is illustrated as follows (Figure 1).

According to Petocz, this continuum covers most of the meanings attributed to the word *symbol*, and psychoanalytic usages can be found across all five of these categories (Enckell, 2010). It is not our intention here to examine the relationship between this continuum and the specific psychoanalysts' usage. Rather, we wish to raise your attention to the narrowest end of the spectrum, as it reflects one of the most characteristic contributions of Freud's work and psychoanalytic discourse.

Actually, at this narrowest level, two types of Freudian symbols are incorporated—what Petocz (1999, p. 24) designates as the “Freudian Broad” (FB) position and “Freudian Narrow” (FN) position. Although both positions can be regarded as unconsciously produced substitute, they differ fundamentally in Freud's original assumptions regarding their formation processes: the former is ontogenetic, whereas the latter is phylogenetic. Laplanche and Pontalis offer a simple and precise differentiation of Freudian symbolism, defining it as follows:

I. Speaking broadly: mode of indirect and figurative representation of an unconscious idea, conflict or wish. In this sense, one may in psycho-analysis hold any substitutive formation to be symbolic.
II. In a more restricted sense: mode of representation distinguished chiefly by the constancy of the relationship between the symbol and what it symbolizes in the unconscious. This constancy is found not only in the same individual and from one individual to the next, but also in the most varied spheres (myth, religion, folklore, language, etc.), and in the most widely separated cultures (Laplanche and Pontalis, 1973, p. 442).

In this paper, we prefer to refer to the former as *individual symbol* and the latter as *universal symbol*, as these terms more clearly convey the distinction between them.^1^

They further observe that this distinction is frequently overlooked in contemporary psychoanalytic discourse: “Today this contrast would appear to have lost some of its clarity in common psycho-analytic usage” (1973, p. 443). Enckell (2010) also notes this phenomenon: Freud expresses a retrogressive orientation on symbolism (whether individual or universal), whereas the post-Freudians (such as Winnicott) stress more on the progressive pattern. Enckell characterizes the former as “archaeology”, where the “symbol” is regarded as regressive, defensive and substitutive, in contrast to the latter “teleology” of “metaphor”, which includes a progressive and creative direction.^2^

This phenomenon in psychoanalytic writings is largely attributable to the post-Freudian shift from “symbol” to “symbolization” (Rodrigué, 1956; Enckell, 2010; Petocz, 2019). In this context, psychoanalysts become increasingly concerned with questions of mental capacity—for instance, the differentiation between presentational symbolization and discursive symbolization (Langer, 1942; Pestalozzi, 2003; Niedecken, 2016). As Petocz pinpoints:

The received view is that throughout the history of psychoanalysis there have been significant changes in how the field has dealt with symbols, with one of the most fruitful being the evolving conception of symbolization…... We have shifted from symbolism to symbolization. (2019, p. 258)

This shift combines with, and reinforces, a general reluctance to engage with Freudian universal symbols. The prevailing tendency of research on symbolism in psychoanalysis is aptly captured in Éric Smadja's observation:

After Freud and his disciples, symbolism and the processes of individual symbolization were explored and studied by the post-Freudian, notably in the field of the psychoanalysis of children with Melanie Klein, Hanna Segal and Donald Woods Winnicott, in particular, and in that of psychoses. However, those working on the subject do not seem to have pursed their investigation of the collective dimension of symbolism which had been quite developed by the pioneering generation. (Smadja, 2019, p. 99)

The neglect of the collective dimension of symbolism, or of universal symbols, offers no advantage for the dialogue between psychoanalysis and other humanities, such as cultural anthropology, folk psychology, and semiotics. Without this dimension, symbolism risks losing its capacity to function as an intermediary and mediating link between the individual and collective fields—a role that is essential in psychoanalysis. Although Freud's principal discovery lies in the symptomatic meaning of symbols and its resemblance to the dream, he consistently regards the bridging of individual psychology and group psychology as part of his intellectual mission. Even in his final published work, *Moses and Monotheism* (Freud, 1939), Freud persistently invokes to transgenerational symbols and archaic inheritance to pursue this aim.

Freud remarks that “The best way of understanding psycho-analysis is still by tracing its origin and development” (Freud, 1923, p. 235). Accordingly, our aim is to present the history of the formation of universal symbols in the works and contributions of Freud and his disciples, primarily Stekel, Abraham, and Jones. This history spans nearly a decade, starting from 1909, when these earliest psychoanalysts prepare to establish a committee for collecting universal symbols, and falling silent in 1917, after Jones' (1916/1948) integrative paper on symbolism and Freud's final discussion of dream symbols in the *Introductory Lecture on Psycho-Analysis* (1915–1917). This proposed historical focus includes what Petocz (1999, pp. 98–124) labels the “core years for the FN theory (1914–1917)”, but it adds considerable extra historical material which sheds additional light on the problem of universal symbols.

## 2 The germination of universal symbols

Freud's interest in symbolism begins before the emergence of psychoanalysis. In his work from the neurological period, Freud (1891/1953), he deals extensively with symbolic issues and divides speech disorder into two types: verbal aphasia and asymbolic aphasia. In this text, the symbol mainly refers to linguistic one, a common usage that denotes the relationship between a word and the idea of a certain object. Although this usage does not impose a restrictive connotation on symbolism, it is sufficient to confirm Freud's early interest in symbolic phenomena.

Between 1893 and 1908, Freud's attention to symbolism is primarily directed toward the symbolic character of symptoms. The terms he frequently employs during this period are *mnemic symbol*,^3^ referring to the substitution of repressed ideas, and *symbolization*, denoting the tendency of hysterics to transform verbal expressions into physical manifestations. Both of these forms of symbolism originate in the memories of subjects' life events and follow the logic of Conflict-Repression-Substitution (Petocz, 1999, p. 46). They therefore fall within the category of individual symbol. However, considerations of universal symbol are already implicit in the first edition of *The Interpretation of Dreams* (Freud, 1900), which we summarize under three aspects:

1) A critical continuation of earlier symbolic methods of dream-analysis, primarily drawing on popular culture and Scherner's theory of imagination. Popular culture has long been fascinated by dreams, often attributing to them specific meanings and even predictive power. In antiquity, the interpretation of dreams is the prerogative of the priest. For example, Aristander's interpretation of a dancing Satyr—the forest god—in the dream of Alexander the Great employs an anagram: he divides Satyros into its homophonic equivalent sa Tyros, meaning not “Satyr” but “Tyre [the city] is yours”. On this basis, the dream is understood to mean that the Satyr is celebrating the city's conquest and that Alexander will be victorious.^4^

Two folk methods of dream-analysis appear in Freud's discussion: the ancient symbolic method and the decoding method. In the former, the dream content is treated as a whole, and interpretation consists in replacing this whole with another, intelligible element that bears some resemblance to it; in the latter, the manifest dream symbols are translated into other fixed and comprehensible significations, which are then reassembled to form an interpretation. Although these two methods differ in quality and nomenclature, they share a common principle: the substitution of unknown and ambiguous dream images with comprehensible and recognizable meanings. In this sense, both folk methods are essentially forms of symbolism, the difference lying only in whether the dream is viewed holistically as a single symbol or whether its variety fragments are treated as different symbols. Freud's criticism of folk symbolism is not aimed at rejecting the tendency to replace chaotic images with accessible meanings, but rather at reconstructing its methodological foundation. His purpose is the same as that of folk methods—assigning meaning to individual dreams—except that he seeks to accomplish this scientifically. The symbolic method depends on the interpreter's talent and intuition, which are not easily generalizable or transmissible, while the decoding method relies primarily on the reliability of “decoding manual”. For these reasons, Freud considers both methods inconsistent with his scientific ideals and therefore methodologically unsound.

In his scientific examination of dream symbolism, Freud primarily engages with Scherner's theory, even acknowledging Scherner in a footnote added to the third edition of *The Interpretation of Dreams* (1911b, p. 359) as the true discoverer of dream symbolism. Scherner posits that the dynamics of dreams arise from the imagination, which symbolizes the activities of the organs and the body during sleep. He states:

(The sexual stimulation dream) derives its name from nerve stimulation of the sex or reproductive organs during sleep. Very characteristic for both male and female sleepers, it appears sometimes independently, that is as a result of self-induced excitement of the highly charged sex organs, and sometimes, in fact most commonly, in connection with the urinary urge and its dream formations, because the physically involved organs stand in the closest interrelationship with one another. The fantasy merely takes the sexual vitality within the physical organ as its motive and represents it symbolically (Scherner, 1861/1992, p. 347).

Some of Freud's later symbolic interpretations closely resemble Scherner's views—for instance, the pipe as a symbol of the penis, and the house as a symbol of the body. Nevertheless, Freud still opposes Scherner's approach, contending that his theory is so imaginative that it reopens the door to random and arbitrary explanations and fails to account for why night dreams only reflect the activities of only certain parts of the body rather than all active organs. Consequently, Freud concludes: “Its lack of any technique of interpreting that can be grasped scientifically must greatly narrow the application of Scherner's theory” (Freud, 1900, p. 226).

2) The role of symbols in the consideration of representability. The mechanisms of dream-work—namely condensation, displacement, consideration of representability, and secondary revision—are the processes through which latent thoughts are transformed into manifest content. The third mechanism, consideration of representability, refers to the selection of psychical materials suitable for expression, particularly the transformation of thoughts and ideas into visual image. This transformation produces two salient characteristics: first, the cancellation of linguistic logic, since logical conjunctions cannot be rendered into concrete image; and second, the abundance of visual elements in dreams. Symbols contribute decisively to this abundance, as they enable the expression of abstract ideas through image. Freud even concludes his discussion of the chapter “Consideration of Representability” with an explicit commendation of symbolism:

There is no necessity to assume that any peculiar symbolizing activity of the mind is operating in the dream-work, but that dreams make use of any symbolizations which are already present in unconscious thinking, because they fit in better with the requirements of dream-construction on account of their representability and also because as a rule they escape censorship. (Freud, 1900, p. 349)

This statement not only accounts for the abundance of symbols in dreams but also indicates that such symbols are not produced by the dream-work itself. If the dream does not create symbols, where do they originate? At this point, Freud leaves open a crucial question concerning the nature and source of universal symbols.

3) Some typical dreams. In 1900, Freud does not yet conduct a systematic investigation of universal symbols; instead, he refers only to certain *typical dreams*. These dreams are common to almost everyone's experience, and, as he observes, “If we attempt to interpret a typical dream, the dreamer fails as a rule to produce the associations which would in other cases have led us to understand it, or else his associations become obscure and insufficient so that we cannot solve our problem with their help” (Freud, 1900, p. 241). These typical dreams can be regarded as precursors to Freudian universal symbols, a connection Freud himself makes when, in 1914, he titles a chapter “Representation by Symbols in Dreams—Some Further Typical Dreams”. Thus, even in the first edition of *The Interpretation of Dreams*, Freud identifies certain trans-individual symbols, whose presentation, meanings, and material sources remain consistent across different dreamers. Owing to the limits of his experience at the time, however, he confines the scope of such dreams to a narrow set—namely, nakedness, the death of the fond person, examinations, and flying. Despite this restricted range, these typical dreams serve as a catalyst for the subsequent flourishing of research on symbolism; indeed, within less than a decade, a wealth of new material and analysis would detonate this long- buried charge.

## 3 Dreams and myths: steps toward universal symbolism

As mentioned earlier, prior to 1909, Freud's work is primarily focused on individual symbolism such as the formation of symptoms, and he limits his interest in and ambition for universal symbolism, presenting it only as a phenomenon. Petocz (1999, p. 57–59) summarizes Freud's shift toward universal symbolism, and we follow her recapitulation and present it as Table 1, which vividly illustrates Freud's cautious attitude and the factors that facilitate his transformation.^5^

**Table 1:** Freud's steps toward universal symbolism.

Step 1	Several symbolic elements exist in dreams, and translations of these elements help in understanding dream contents. Similar symbols are found in myths, folklore, legends, and popular culture.
Step 2	Psychoanalysts show increasing interest in mythology and linguistics. Wilhelm Stekel and others begin to search for and investigate symbolism, mainly to discover new symbols and provide translations of them.
Step 3	Freud hesitates on this issue. On the one hand, independent research on symbolism is advancing and proving fruitful; on the other hand, Freud realizes that such research is at risk of regressing toward the ancient symbolic method.
Step 4	Freud's interest in symbolism increases, and he recognizes the inevitable necessity of addressing the universality of symbols and the implications of symbolism for psychoanalytic theories.
Step 5	Freud acknowledges that universality in symbols involves fixity (constant meanings) and muteness (failure of associations). Their meanings can be scientifically ascertained via relevant evidence from the cultural fields of myth, ritual, fairy tale, and so on.
Step 6	Freud argues that universal symbols are acquired in a way distinct from other unconscious ideas and are connected to the ancient language. Ultimately, he claims that symbols derive from a phylogenetic source.

This table outlines the evolution of Freud's theory of universal symbols. In 1908, Freud is already exploring mythological and philological materials to demonstrate the universality of symbols in neurotic symptoms. When constructing the symbolic relationship between money and feces, he comments:

In reality, wherever archaic modes of thought have predominated or persist—in the ancient civilizations, in myths, fairy tales and superstitions, in unconscious thinking, in dreams and in neuroses—money is brought into the most intimate relationship with dirt……Indeed, even according to ancient Babylonian doctrine gold is ‘the feces of Hell' (Mammon=ilu manman). Thus in following the usage of language, neurosis, here as elsewhere, is taking words in their original, significant sense, and where it appears to be using a word figuratively it is usually simply restoring its old meaning. (1908, p. 174)

The tendency to incorporate mythology and linguistics into psychoanalysis has intensified since 1909, and Freud even contemplates publishing a psychoanalytical dream book:

When we have become familiar with the abundant use made of symbolism for representing sexual material in dreams, the question is bound to arise of whether many of these symbols do not occur with a permanently fixed meaning, like the ‘grammalogues' in shorthand; and we shall feel tempted to draw up a new ‘dream-book' on the decoding principle. On that point there is this to be said: this symbolism is not peculiar to dreams, but is characteristic of unconscious ideation, in particular among the people, and it is to be found in folklore, and in popular myths, legends, linguistic idioms, proverbial wisdom and current jokes, to a more complete extent than in dreams. (Freud, 1909, p. 351)

Why does Freud's curiosity about universal symbols intensify in 1909? To address this question, it is necessary to examine the history of the early psychoanalytic circle and to explore the relevant context. We intend to highlight two primary reasons. First, the contributions of Freud's disciples—particularly Abraham, Stekel, and Jung—demonstrate the correlation between dreams and myths, as well as the compatibility of psychoanalysis with folk psychology. Freud's correspondence with his colleagues during this period extensively reflects their exchanges and developing ideas on symbolism. Second, Freud's theoretical ambitions and the growing dissemination of psychoanalysis naturally compel him to engage with broader intellectual issues. In 1909, he is invited by Stanley Hall to attend the anniversary celebration of Clark University, where he delivers five lectures in German. His comments on this trip reveal his aspirations for psychoanalysis. In his honorary degree speech, he proudly refers to this occasion as “the first public recognition of our efforts” (Gay, 1988, p. 266). From his solitary beginnings to the establishment of the Wednesday Psychology Society in the autumn of 1902, and subsequently to the lectern at Clark University, the influence of psychoanalysis steadily intensifies. As its influence expands, psychoanalysis inevitably confronts certain obstacles: overcoming the geographical and intellectual limits of the Viennese circle, avoiding the label of “Jewish psychology”,^6^ and extending the applicability of its findings. Given that psychoanalytic clinical practice cannot be directly demonstrated to the public or replicated experimentally, cultural and linguistic materials emerge as the most suitable vehicles for justifying and universalizing psychoanalytic conclusions.^7^

Freud's works after 1909 increasingly incorporate themes relating to mythology and folklore, such as the antithetical meaning of primal words (1910), dreams in folklore (Freud and Oppenheim, 1911), totemism in primitive tribes (Freud, 1913), and the parallels between delusions and myths in the Schreber case (Freud, 1911a). These investigations substantially facilitate the progression of psychoanalytic symbolism, since symbols function as essential components that bridge the individual psychology and folk psychology.

## 4 Mythology and linguistics: two foundations of universal symbolism

At the beginning of 1910, Freud and Jung almost simultaneously shift their research focus to mythology and notice the intimate correlation between folk elements and symbolism. In a letter to Freud dated January 30, 1910, Jung reports that:

I had two public lectures this week. ……The (one) subject was “symbolism.” I have worked at it and tried to put the “symbolic” on a psychogenetic foundation, i.e., to show that in the individual fantasy the *primum movens*, the individual conflict, material or form (whichever you prefer), is mythic, or mythologically typical. [Jung, 1910, Jan. 30] (McGuire, 1974, p. 288, 289; italics originally)

Freud's response is:

Your deepened view of symbolism has all my sympathy. Perhaps you remember how dissatisfied I was when in agreement with Bleuler all you had to say of symbolism was that it was a kind of “unclear thinking.” True, what you write about it now is only a hint, but in a direction where I too am searching, namely, *archaic regression*, which I hope to master through mythology and the *development of language*. (Freud, 1910, Feb. 2) (McGuire, 1974, p. 291; italics originally)

Freud does not directly claim that symbolism is related to mythology. For him, symbolism belongs to a broader domain—the archaic remnants in the mind—and he treats mythology and diachronic linguistics as avenues for exploring this domain. Consequently, he does not produce a systematic account of symbolism until 1914; instead, he addresses various “primitive” and “archaic” issues. It is his colleagues who spark the interest in symbolism and help to promote its flourishing within psychoanalysis.

Stekel makes several debatable contributions to symbolism during this period. Freud candidly acknowledges his work in the preface to the third edition of *The Interpretation of Dreams* in 1911:

The theory of dream interpretation has itself developed further in a direction on which insufficient stress had been laid in the first edition of this book. My own experience, as well as the works of Stekel and others, have since taught me to form a truer estimate of the extent and importance of symbolism in dreams (or rather in unconscious thinking). Thus in the course of these years much has accumulated which demands attention. (Freud, 1911b, p. xxvii)

Stekel's contributions to symbolism consist of two parts: first, at the Nuremberg Conference in 1910, he proposes establishing a committee within the psychoanalytic organizations to collect various symbols^8^; second, he publishes his personal treatise on symbolism, *The Language of Dreams* (*Die Sprache des Traumes*). Stekel demonstrates a strong intuition and sensitivity to the unconscious symbolism, at times even surpassing Freud. As Jones states in his official biography:

Stekel was a naturally gifted psychologist with an unusual flair for detecting repressed material, and his contribution to our knowledge of symbolism, a field in which he had more intuitive genius than Freud, were in the earlier stages of psycho-analysis of very considerable value. Freud freely admitted this. He said he had often contradicted Stekel's interpretation of a given symbol only to find on further study that Stekel had been right the first time. (Jones, 1955, p. 151, 152)

Before the Nuremberg Conference, Jones sends Freud a suggestive letter:

Do you not think the time is ripe to apply a suggestion you made in the *Traumdeutung*, namely to make a collection of typical dreams? Why not establish a central bureau at Jung's to which short accounts of analyses could be sent by different workers? [Jones, 1910, Feb. 12] (Bos and Roazen, 2007, p. 69)

Freud adopts this suggestion and asks Stekel to present it before the Congress. Ultimately, a committee for collecting symbols is established, consisting of Abraham, Maeder, and Stekel.

*Die Sprache des Traumes*, published in 1911, constitutes Stekel's major contribution to the discussion of symbolism; however, it diverges sharply from Jones' (also Freud's) conception. For Jones, the study of symbolism should be pursued through the comparative investigations of dreams, jokes, and folk elements such as myths and idioms, whereas Stekel disregards this paradigm and instead relies heavily on his intuition. The book receives substantial criticism, including from Freud himself. In his autobiography, Stekel writes with grievance:

My book on dreams was published in 1911. I had discovered many dream symbols and explained the symbolism of death in several chapters. One evening was set aside in our group for the discussion of this book. I did not expect acknowledgment, for I had already suffered bitter disappointments. ……I expected acknowledgment from Freud, but even he failed me. (Stekel, 1950/2013, p. 73, 74)

In fact, Freud's attitude toward Stekel's approach to symbolism is not altered after the publication of this book; rather, he has already harbored a negative impression of Stekel's research before the conference. His critiques of Stekel are scattered across various pieces of correspondence, and in two letters to Jones and Jung in 1909, he states:

He (Stekel) is weak in theory and thought but he has a good flair for the meaning of the hidden and unconscious. His book cannot satisfy me personally, but it will do immensely good among the outsiders, his level being so very much nearer to theirs. I am glad you like Abraham's work far better; he is a sharp thinker and has set his foot on fertile ground. (Freud, 1909, Nov. 20) (Jones, 1955, p. 69)

A book on dream symbols doesn't strike me as impossible, but I am sure we shall object to the way Stekel goes about it. He will work haphazardly, taking whatever he can lay hands on without regard for the context, and without taking myth or language or linguistic development into account. (Freud, 1909, Nov. 21) (McGuire, 1974, p. 266)

Therefore, the psychoanalytic community's evaluation of the style of *Die Sprache des Traumes* is far from accidental. Stekel reintroduces into psychoanalysis the intuitive interpretation of dreams that Freud had previously rejected, and he inverts Freud's basic structure of dreams. He argues that the manifest content already conveys a dream's meaning, thereby freeing psychoanalysts from the meticulous work of uncovering latent thoughts. Stekel's intuition about the unconscious thus functions both as the driving force behind his explorations and as the very factor that plunges him into the abyss of intuitionism.

Unlike Stekel, Abraham's work aligns more closely with Freud's preference for mythology and linguistics. In 1909, Abraham publishes a seminal study in mythological research, *Dreams and Myths: A Study in Folk-Psychology*. In this work, he explicitly states the aims of mythological studies and highlights the connection between his research and Freud's theories:

In addition to the products of individual phantasy, however, there are phantasies not to be ascribed to any one individual. Myths and fairy-tales are formations of this kind. We do not know who created them, nor who first recounted them. They were handed down from generation to generation, undergoing many additions and changes in the process. It is in legends and fairy-tales that the phantasy of a nation is revealed.……The present monograph is an attempt to compare myths with the phenomena of individual psychology and especially with dreams. Its purpose is to demonstrate that Freud's doctrines can to a large extent be applied to the psychology of myths and so provide a new basis for their understanding. (Abraham, 1909/1955, p. 154)

This study accords closely with Freud's conception of symbolism. Abraham undertakes a comparative analysis of symbols in mythology, language, and dreams, and defends the legitimacy of sexual symbols that have been criticized by the scientific community. Evidence from diverse forms of human fantasy and language suggests that symbols are deeply engrained in the human psyche and frequently sexual in nature. For instance, the snake functions as a symbol of the male genital—a motif recurring in myths across cultures, including the Bible, Greek mythology, Norse mythology, and German fairy tales (Riklin, 1908/2016). Likewise, linguistic usage bears witness to sexual symbolism:

Even more remarkable is the fact that in all languages inanimate objects are sexualized. In various languages a particular gender is regularly or at any rate preferably allotted to a given object. This must be due to a sexual symbolism prevailing among different peoples. (Abraham, 1909/1955, p. 164)

In summary, 1909 is a pivotal year in the history of psychoanalytic research on universal symbolism. The connection between mythology, linguistics, and symbols becomes increasingly evident, extending the scope of symbolism from individual phenomena—such as symptoms and parapraxes—to more universal topics, including nations and cultures. Symbolism serves as a bridge between psychoanalytic symptomatology and cultural studies, and the significance of symbols shares similarities at the individual and collective levels. This is undoubtedly an exciting discovery and continues to inspire enthusiasm within the psychoanalytic community, as symbolism not only facilitates the expansion of psychoanalytic research but also helps dispel the skepticism about the validity of psychoanalytic knowledge derived from patients. From Freud's praise and criticism of his colleagues' work on symbolism during this period, as well as from internal discussions within the central Freudian group, it is evident that Freudian universal symbolism does not rely on the personal talent or intuition of psychoanalysts but on the comparative study of folk psychology (Völkerpsychologie in Wundt's sense). Through symbolism, psychoanalysis transcends the confines of purely individual psychology and engages with the broader realm of collective culture.

## 5 Culture or individual: the genesis of universal symbols

Freud does not devote a separate section to the topic of symbolism until the fourth edition of *The Interpretation of Dreams* in 1914, yet he is already engaged in the debate on symbolism in other forms. In his commentary on the minutes of the Vienna Psychoanalytic Congress of November 10, 1909, he asserts: “Dream symbols that did not find a similar corroboration in myth, fairy-tale, popular custom, etc., would have to be [regarded as] questionable” (Nunberg, 1967, p. 311). During this period, Freud increasingly directs his attention to the evolution of language. Referring to a booklet by linguist Abel, he elaborates on the importance of ancient linguistic expressions in the analysis of dreams. Abel observes that in ancient Egyptian, words can simultaneously express opposite meanings or ideas—an observation that mirrors Freud's earlier discovery that contradictory images can appear in a single dream. This parallel between dreams and the oldest languages pushes Freud to renew his appreciation for the archaic elements embedded in dreams:

In the correspondence between the peculiarity of the dream-work mentioned at the beginning of the paper and the practice discovered by philology in the oldest languages, we may see a confirmation of the view we have formed about the regressive, archaic character of the expression of thoughts in dreams. And we psychiatrists cannot escape the suspicion that we should be better at understanding and translating the language of dreams if we knew more about the development of language. (Freud, 1910, p. 161)

This serves as a direct prelude to his formulation of the genesis of symbols 4 years later:

Things that are symbolically connected today were probably united in prehistoric times by conceptual and linguistic identity. The symbolic relation seems to be a relic and a mark of former identity. In this connection we may observe how in a number of cases the use of a common symbol extends further than the use of a common language. (Freud, 1914b, p. 352)

Thus, symbolism becomes closely linked to diachronic linguistics, and the use of cross-linguistic similarities to substantiate the symbolic meanings identified by psychoanalysis emerges as a new “royal road”. Ironically, Ferenczi (1908/1994) draws on a vulgar Hungarian expression to prove that guns are phallic symbols, while ignoring the more immediate resemblance in their external appearances.

Using language and myth as evidence for universal symbols inevitably introduces a paradigm tension, as psychoanalytic material consists primarily of private symbols elicited in the consulting room. Although, as mentioned earlier, psychoanalysts around 1909 display tremendous curiosity about the cultural dimensions of symbols, clinical practice compels them to preserve the individual aspect of psychoanalytic symbolism. This tension crystallizes into a fundamental question: Do symbols originate in the individual or in the collective? Should psychoanalysts interpret them from an individual perspective or from a cultural perspective? Despite the flourishing discussions of symbols, two divergent perspectives continued to coexist within the first psychoanalytic group, rendering its position on symbolism vacillating and uncertain.

The primary task of psychoanalysts who adopt an individual perspective is to delineate how symbols emerge from a person's psychological operations—namely, the ontogenesis of symbols. Two main approaches are available: one is based on Freud's early ideas about symbolic mechanisms, in which conflict, repression, and substitution are vital to symbol formation; the other explains symbolic equivalence in terms of the pleasure principle and reality principle, treating the symbol as a product of the interaction between the mind and reality. Ferenczi formulates the first approach in his 1913 work *The Ontogenesis of Symbols*. In his view, psychoanalytic symbols refer to objects or ideas that are cathected with intense affect that are logically incomprehensible and without apparent cause, while their true source lies in the unconscious. Equivalence—i.e., A is identified with B under condition X—is usually regarded as the basis of a symbolic relationship. But, for Ferenczi, A=B (under condition X) is a figurative structure, and “not all similes, therefore, are symbols, but only those in which the one member of the equation is repressed into the unconscious” (Ferenczi, 1913/1994, p. 277, 278). Psychoanalytic symbols constitute an equivalence between a conscious element and an unconscious one, and it is the individual's emotions and motivations, rather than rational conditions, that produce this equivalence. Symbolic relationships are primarily built on an individual's subjective associations, while objective characteristics merely provide the conditions for their establishment. The second approach is elaborated by Jones in his integrative paper on symbolism. Jones (1916/1948, p. 107) posits three elements that give rise to symbolic equivalence: mental incapacity, the pleasure-pain principle, and the reality principle. For Jones, the dynamics of equivalence is rooted in the mind's adherence to the latter two fundamental principles. In our view, both of these principles in Jones' account ultimately derive from the mind's *inertia*—a tendency to regulate external experiences either by incorporating and assimilating these experiences or by projecting internal schemas to facilitate understanding of the outside world. Combining the ideas of Ferenczi and Jones, the ontogenesis of symbols is described as follows: the individual mind's capacity to establish equivalence provides the conditions for symbol formation; only when that equivalent relationship is regulated by one's affect or motivation that represses one end of the equation does a true symbol form.^9^

By contrast, Rank and Sachs define six characteristics of true psychoanalytic symbols: Representation of the unconscious, constant meaning, independence of individual conditions, evolutionary foundations, speech relationships, phylogenetic parallels (in myths, cult, religion, etc.) (Rank and Sachs, 1916, p. 22). Except for the first characteristic, they all reflect the cultural, evolutionary and historical aspects of symbols. In fact, if we focus on the third characteristic in particular, symbols exist as exterior or heterogeneous entities for the individual. It is impossible for the individual to provide any personal associations where such symbols appear, and the subject's speech cannot reach the symbolic realm. This is what Freud describes as “mute” of symbols. The definition offered by Rank and Sachs suggests that collective elements such as culture hold a dominant position in symbolism. Freud partly admits this cultural dominance when he remarks that symbolic connections are never acquired through learning but are phylogenetic heritages (Freud, 1915–1917, p. 199).

At this time, there is also an inclination within psychoanalysis, represented by Jung, to restore the ancient heritage of symbolism and eliminate its individual components. As Forrester observes:

The difference between myth analysis and dream analysis consisted in the absence of “individual association”. Since this was the methodological point of entry of symbolic decoding of dreams, myth analysis had this in common with interpretation using symbols. Jung certainly equated myths and symbols precisely because of this characteristic: he never paid as much attention to individual associations as Freud did. (1980, p. 101)

This tendency is evident in Jung's 1912 publication *The Transformations and Symbols of the Libido*. His interest in mythology and symbolism carries a distinct flavor of mysticism. In his view, incestuous impulses and the Oedipus complex are symbols of a higher-level idea—one that is typically obscure, mysterious, and prophetic. In *On the History of the Psycho-Analytic Movement*, Freud points out that Jung's idea totally ignores the differentiation between the symbolic and the real, thereby denying the reality of the unconscious:

When Jung tells us that the incest-complex is merely “symbolic”, that after all it has no “real” existence, that after all a savage feels no desire toward an old hag but prefers a young and pretty woman, we are tempted to conclude that “symbolic” and “without real existence” simply mean something which, in virtue of its manifestations and pathogenic effects, is described by psycho-analysis as “existing unconsciously”—a description that disposes of the apparent contradiction. (Freud, 1914a, p. 64)

Jung's interest in the phylogenesis of symbols is closely connected to his research on psychosis, a field in which Freud has little direct experience. In a letter to Freud, Jung firmly asserts: “In my view the concept of libido as set forth in the Three *Essays* needs to be supplemented by the genetic factor to make it applicable to Dem. praec (Dementia praecox)” [Jung, 1911, Nov. 14] (McGuire, 1974, p. 461). In Jung's theory, symbols originate entirely from the collective unconscious; The subject has no experiential access to them and cannot conceive of any knowledge about them (Settineri et al., 2017). Freud rejects both Jung's disregard for the reality of the unconscious and his mystical orientation. He insists that psychoanalytic research must proceed on a scientific basis.

It is clear from all of this material that the debates over the ontogenesis vs. the phylogenesis of symbols raise a series of questions. For instance, can individuals actively generate symbolic connections? For individual symbols, the answers are relatively straightforward and affirmative; but for universal symbols, they are more complex—particularly when the question is reformulated as, “can individuals create universal symbolic connections?” Although Freud regards symbolic connections as a phylogenetic legacy, he does not adopt an entirely negative stance toward this possibility. Drawing on the views articulated in Jones' comprehensive article, the debate can be addressed as follows: first, the interpretation of symbols is not arbitrary; yet this does not imply that each symbol corresponds to a single fixed meaning. A symbol may hold different meanings across individuals, dreams, and myths, but the range of such meanings is limited to a specific set. Second, individuals can create new symbolic connections. But these creations conform to certain principles that are themselves universal, such as the consistency of perception. Finally, different symbols may be chosen to convey an identical idea—for example, the male genital may be symbolized by diverse images such as the cock or the snake. These answers are examined in detail in Petocz's (1999) work and can find support in Freud's own writings. The perceptual consistency of experience, together with common biopsychosocial conditions, provides the foundation for universal symbols. The additional historical material presented above demonstrates that even when Freud is compelled to acknowledge the phylogenetic sources of universal symbols, he remains reluctant to accept the methodologies of Jung and Stekel. For Freud, a scientific approach is imperative. Consequently, neither Stekel's intuition nor Jung's mysticism is acceptable for true psychoanalysis. Such an intuitive-mystical orientation will unavoidably revert symbolism to the ancient method, which dismisses the analysand's discourse and privileges the analyst's function and authority.

## 6 Conclusion

Freudian universal symbols rest on an evolutionary presupposition—namely, that certain phylogenetic inheritances from ancient times persist in the unconscious. Although Freud does not formulate a notion of the “collective unconscious” in the manner of Jung, he shows a clear interest in the archaic components of the mind. The archaic elements of universal symbols are regarded as remnants of the origin and evolution of language, which offers a scientific basis for the formation of symbolic connections. Freud (1915–1917, p. 145, 146) cites the linguist Sperber to account for the predominance of sexual contents in dream symbols. According to Sperber, speech sounds are initially employed for communication and for attracting sexual partners; over time, the sexual connotations of words gradually detach from their original referents and shift toward other communicative functions.

However, symbols are not only confined to linguistic effects; they also transcend language. For example, when snakes function as symbols of male genitals, it is clearly that this link arises from perceptual resemblance. In this respect, we prefer to regard symbols in classical psychoanalysis as what Peirce (1931, p. 276) calls “icons”, i.e., signs that bear a qualitative connection to their objects in the process of signification. Many symbolic connections discussed by Freud—hats symbolizing male genitals, emperors and empresses symbolizing fathers and mothers, hollow objects such as cabinets and stoves symbolizing the womb, and climbing stairs symbolizing sexual intercourse—display a concrete correspondence between the symbol and its referent. The coherence of these connections is often intuitively apprehensible without recourse to linguistic-evolutionary explanations. Accordingly, we agree with Petocz's (1999) statements which ground the origin of universal symbols in the perceptual consistency of external objects, with language serving merely as a medium for articulating such consistency. In other words, perception is primary, and language is secondary.

After 1917, psychoanalytic interests in symbolism appears to wane, and related discussions largely stagnate. On the one hand, as Forrester (1980) observes, psychoanalytic research begins to shift toward sociological paradigms, as societal factors push psychoanalysis away from historical and archaic concerns and facilitate it toward more pragmatic reality issues. Freud's postulation of the death drive and his formulation of the second topographical model develop in response to the reality at that time, particularly the traumatic experience of World War I. On the other hand, the departures of Stekel and Jung from the psychoanalytic circle remove two figures whose work has heavily sustained interest in symbolism. The debates of 1909–1917 have already established the basic principles of the orthodox psychoanalytic approach to symbolism. Consequently, for the following decade, psychoanalytic theories of symbolism remain largely dormant, with only a few scattered contributions appearing in the literature.^10^

In our view, Freudian universal symbols confront a fundamental difficulty: if such symbols are indeed phylogenetic heritages operating within the unconscious, then Freud is implicitly affirming the possibility of transgenerational transmission of psychological content. This position, essentially Lamarckian in nature, have already been rejected by the scientific community, and by Freud's time no serious biologist would have defended Lamarck's claims (Obaid and Fabián, 2022). Freud's proposal to investigate mythology and linguistics in order to support psychoanalytic findings on symbolism is, without doubt, a compelling way to link psychoanalysis with other human sciences; yet it is neither necessary nor logically inevitable to assume that symbols derive from archaic remnants. Lacan (1953/2006) has demonstrated with great precision that the effectiveness of symbols arises from the function of the signifier, which allows us to examine Freudian universal symbols without presupposing the inheritance of ancestral experience. However, even if we can exclude the phylogenetic supposition in the Freudian universal symbol, the archaeological background still prevents it from obtaining full recognition in the contemporary psychoanalytic climate. Although Petocz (2019, p. 255–280) offers a response to this issue, a persistent question raised by Enckell's (2010) classification remains: if universal symbolism and its promise for bridging psychoanalysis and wider cultural disciplines is to be given the place it deserves, how will that negotiate the contemporary move from the “archaeological” to the “teleological”?

This question requires further reflection and investigation. Here, we just emphasize the significance of the Freudian universal symbol by quoting Lacan's statement in his most renowned article:

For Freud's discovery was that of the field of the effects, in man's nature, of his relations to the symbolic order and the fact that their meaning goes all the way back to the most radical instances of symbolization in being. To ignore the symbolic order is to condemn Freud's discovery to forgetting and analytic experience to ruin. (1953, p. 227)

If we abandon the exploration of Freudian universal symbols, we forfeit not only the dialogue between psychoanalysis and other disciplines dedicated to the universality of the human mind, but also a vital part of our concern for human nature.

## Data Availability

The original contributions presented in the study are included in the article/supplementary material, further inquiries can be directed to the corresponding author.

## Footnotes

1 This classification shares certain aspects with Petocz's (1999) attempt to synthesize the FB and the FN position. In her investigation, the presupposition of “system unconscious” is rejected, which means that symbols are not a natural derivation of phylogenetic inheritance. Moreover, the definition provided by Laplanche and Pontalis also reveals that they have already accepted Petocz's views—for they only mention constancy (the same meaning of universality), and not phylogenetic transmission. By dividing the Freudian symbol into *individual symbol* and *universal symbol*, we aim to highlight the broader applicability—not only restricted to the clinical situation—of Freudian symbolism.

2 In previous work, one of the authors (Huo and Chi, 2025)—following Enckell's classification methodology—also identifies two symbolic forms in psychoanalytic literature and attribute the progressive direction to the contributions of (Klein 1930), especially her article *The Importance of Symbol-Formation in the Development of the Ego*.

3 This term first appeared in *The Neuropsychoses of Defence* (Freud, 1894) in the following text: “The conversion may be either total or partial. It proceeds along the line of the motor or sensory innervation which is related—whether intimately or more loosely—to the traumatic experience. By this means the ego succeeds in freeing itself from the contradiction [with which it is confronted]; but instead, it has burdened itself with a *mnemic symbol* which finds a lodgement in consciousness, like a sort of parasite, either in the form of an unresolvable motor innervation or as a constantly recurring hallucinatory sensation, and which persists until a conversion in the opposite direction takes place” (Freud, 1894, p. 49; italic by the authors). (Petocz 1999, p. 36) remarks that the concept of *mnemic symbol* first occurs in *Preliminary Communication*, yet this statement is somewhat ambiguous since the term does not actually appear in that article. A clearer formulation would be: “The concept of what was soon to be termed ‘mnemic symbol' first occurs in *Preliminary Communication* (1893)”. [This is what Petocz should have written in order to be clearer. In other words, the concept occurs in the *Preliminary Communication*, and the term itself appears soon after in *The Neuropsychoses of Defence* (Freud, 1894)].

4 This example is mentioned in the ancient Greek diviner Artemidorus' *Oneirocritica* (Harris-McCoy, 2012, p. 324), which Freud cites in a footnote to the third edition of *The Interpretation of Dreams* in 1911. The interpretive method described here is strikingly familiar within Chinese culture, where *character demolition* (i.e., the deconstruction of written characters) has long been a traditional form of divination. According to the *Origin of Chinese Characters*, every character is formed through one of six methods: Pictograms, Ideograms, Ideogrammic compounds, Phono-semantic compounds, Transformed cognates, and Rebus (Kai, 2015). Dream elements can thus be interpreted on the basis of these compositional principles.

5 For the purpose of this article, we select only Petocz's discussion of universal symbols, that is, the FN position in her book. In her original account, Petocz presents a more diffuse discussion of the converging steps, not only to highlight the convergence onto the FN position but also to reveal the full extent of Freud's uncertainties, vacillations, and competing material that are more conducive to the FB approach—even during the “core years” for the FN theory.

6 Psychoanalysis originates and thrives among a predominantly Jewish community, and Freud always worries about the potential threat of anti-Semitism. In a letter to Abraham, he writes: “I think that we as Jews, if we wish to join in anywhere, must develop a bit of masochism, be ready to suffer some wrong” (Freud, 1908, July 23) (Falzeder, 2002, p. 156).

7 Compared with *The Interpretation of Dreams*, which initially failed to achieve the expected sales, *The Psychopathology of Everyday Life* plays an undeniable role in the spread and acceptance of psychoanalysis. During Freud's lifetime, this book is reprinted eleven times and translated into twelve languages—a popularity matched only by *Introductory Lectures on Psycho-Analysis*.

8 This is particularly crucial to the evolution of psychoanalytic symbolism, as it exposes the political dynamics within the early psychoanalytic circle—dynamics that foreshadowed later divergences and trajectories. The political elements here refer to Freud's designation of Jung as the first president of the International Psychoanalytic Association and the following disputes. Stekel and Adler, as the earliest practitioners of psychoanalysis, play an important role in founding the early psychoanalytic organization (the Wednesday Psychological Society, later renamed the Vienna Psychoanalytic Society). Dissatisfied with Freud's appointment of Jung—a non-Jew—as president, they secretly convene a meeting with several colleagues at Stekel's hotel. In his notes on the history of the analytical movement, Stekel recalls: “I improvised a presentation on symbolism, which linked up with the previous presentations very well and received much approval, inspiring new research into symbolism. A committee was created—but over the next 2 years it did not receive a single submission. That congress was also to become the starting point of a division among analysts. We had already noticed a long time beforehand how Zurich used to ‘manipulate' Freud, turning him against the Viennese followers” (Stekel, 1926/2007, p. 145, 146). To balance and ease the internal political tensions, Freud subsequently assigns the newly established journal *Zentralblatt für psychoanalyse* to Stekel and Adler for editorial management.

9 In discussing the causes of repression, Ferenczi concedes that it is “the result of cultural education” (1913/1994, p. 280). Here, he reintroduces a collective dimension into his ontogenetic formulation.

10 This does not imply that psychoanalytic theory of symbolism is in decline. Discussions concerning the specific meanings of dream symbols still continue (Ferenczi, 1921/2002; Abraham, 1922/1988). Notably, the materials in these scattered studies are drawn almost exclusively from patients' dreams, with relatively little reference to folklore and mythology. It seems that, after the period from 1909 to 1917, psychoanalysis returns to the patient's individual material. This time, however, psychoanalysts are more convinced of the universal applicability of individual material. While symbols are universal and collective, dreams are always individual; thus, the universality of symbols must ultimately be subordinate to the individuality of dreams.
