# Correction: The formation of the Freudian universal symbol: a historical perspective

**DOI:** 10.3389/fpsyg.2026.1994041

**Published:** 2026-09-21

**Authors:** Chuansong Huo, Fei Ju

**Affiliations:** 1 School of Humanity, Tongji University, Shanghai, China; 2 Laboratory of Psychopathology and Psychoanalysis, Graduate School of Human and Environmental Studies, Kyoto University, Kyoto, Japan

**Keywords:** symbolism, psychoanalysis, Freudian symbol, symbol formation, dream-analysis

In the published article, author Chuansong Huo was erroneously assigned as corresponding author. The correct corresponding author is Fei Ju.

The original version of this article has been updated.

